# Electrocardiographic Changes After Suicidal Digoxin Intoxication in a Healthy Woman

**DOI:** 10.2174/1874192401711010058

**Published:** 2017-05-16

**Authors:** Natalia Lorenzo Muñoz, Amparo Benedicto Buendía, Fernando Alfonso Manterola

**Affiliations:** Department of Cardiology, Hospital Universitario de La Princesa, Madrid, Spain

**Keywords:** Digoxin, (CT) scan, Electrocardiogram (ECG), Myocardial necrosis, Multiple arrhythmias

## Abstract

**Background:**

Suicidal digoxin intoxication is a rare clinical entity. Clinical suspicious remains of paramount importance as adequate interpretation of the electrocardiographic changes enable to readily initiate treatment.

**Method:**

We describe a case of suicidal attempt after massive digoxin intake that was satisfactory managed with conservative management strategy that involved a close clinical surveillance of the evolving electrocardiographic changes and digoxin serum levels.

## CASE REPORT

**Fig. (1):**
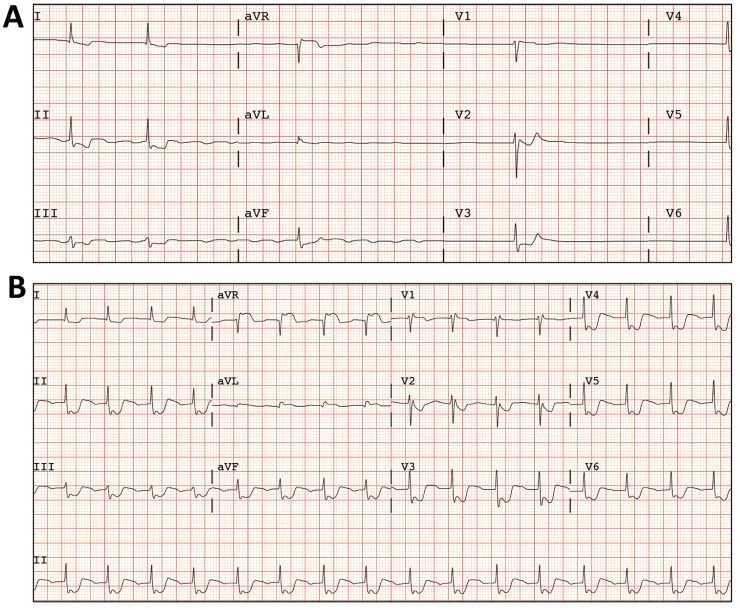
**A**) Atypical atrial flutter alterning with atrial silence. **B**) ECG after Atropin (1mg).

A 53 year-old-woman, nurse, with no previous medical history, was admitted to the emergency unit after polytraumatism during a traffic accident. On admission, the patient was vomiting, uncooperative and with somnolence but conscious and with a Glasgow coma scale score of 15. Blood pressure was normal and her heart rate was 47 bpm. Whole-body computed tomography (CT) scan revealed fracture of the posterior arch of the seventh rib with no other remarkable findings. Blood tests were normal, except from leukocytosis (17.260/mm^3^/mL and elevated creatin-kinase (632 U/L). During initial monitoring, an episode of asymptomatic extreme bradycardia that readily responded to atropine (1mg) was documented. The electrocardiogram (ECG) revealed atypical atrial flutter alternating with atrial silence, with a ventricle response between 25 and 30 bpm (Fig. **1A**). The ECG also demonstrated widespread ST segment depression of 1-2 mm with ST segment elevation in V1 and aVR (Fig. **1A**). Notably, the ST segment changes became even more bizarre during tachycardia (after atropine) (Fig. **1B**). Nevertheless, she denied any chest pain. The enzymatic curve did not suggest myocardial necrosis and the echocardiogram was normal. Although the patient repeatedly denied taking any drugs, eventually toxic analyses were done. Abuse drugs were all negative. However, digoxin serum levels were extremely high (17,93 ng/mL). As the patient remained hemodynamically stable, a conservative medical management under a careful watchful waiting strategy and fluid therapy was selected. Seventy-two hours after admission the patient recovered sinus rhythm (digoxin levels: 10,22 ng/mL) and 5 days after admission she maintained 1:1 conduction (digoxin levels: 3,39 ng/mL). Fig. (**2**) shows ECG changes over time related to the digoxin levels. After 5 days in hospital, the patient finally confessed having acquired 30mg of digoxin (approximately 10 hours before the first determination in blood) and taken the drug with suicide intentions.

## DISCUSSION

Digitalis glycosides have been used for over 100 years and digoxin continues to play a major role in treatment of patients with heart failure and atrial fibrillation. However, digitalis administration has been classically recognized as a well-established cause of iatrogenic morbidity. Importantly, digitalis intoxication remains a common problem in routine clinical practice because its therapeutic window is relatively narrow (from 0.5 to 2 ng/ml). The earliest ECG finding in this scenario is the presence of T-wave changes of virtually any form, ranging from flattening and inversion (that can simulate ischemia or pericarditis) to other abnormal waveforms. At therapeutic levels, digoxin impairs conduction through the AV node but, simultaneously, increase cardiac automaticity, particularly in the Purkinje fibers. The final result is an increased propensity toward automaticity concomitant with a slowing of the AV node conduction. Due to all these effects, multiple arrhythmias have been classically documented with toxic concentrations [1,2].

Digoxin can produce virtually any type of cardiac arrhythmia. Premature ventricular beats are the most common, and often, the earliest arrhythmia associated with digitalis intoxication. Depressed conduction is also a predominant feature of digoxin toxicity [2]. Along with increased ventricular ectopy and conduction block, the three most classical arrhythmias that should immediately suggest digitalis intoxication include: paroxysmal atrial tachycardia with AV block, junctional tachycardia, and ventricular tachycardia [3].

Although suicide with massive digoxin overdose is an exceedingly rare event, there are several cases reported in the literature [4, 5]. In this setting antidigoxin Fab fragments are recommended, as they seem to be safe and effective. However, this strategy is certainly expensive and it has not been supported by clinical trials. Furthermore, the dosage regimen to achieve optimal binding of Fab to digoxin remain to be determined [5].

To the best of our knowledge, there are no previous reports correlating lethal serum leves of digoxin with the natural evolution of the ECG changes over time. Furthermore, our findings suggest that a conservative medical management, with a watchful-waiting strategy under intensive surveillance, is safe and effective in this clinical scenario.

## Figures and Tables

**Fig. (2):**
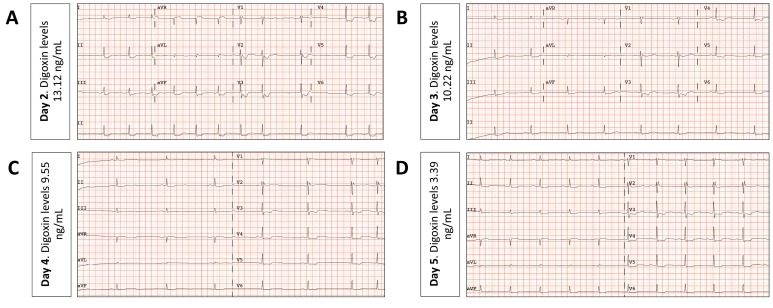
Evolution of ECG changes from day 2 (**A**) to day 5 (**D**) and its correlationship with digoxin serum levels.

## References

[1] Yang E.H., Shah S., Criley J.M. (2012). Digitalis toxicity: A fading but crucial complication to recognize.. Am. J. Med..

[2] Ma G., Brady W.J., Pollack M., Chan T.C. (2001). Electrocardiographic manifestations: Digitalis toxicity.. J. Emerg. Med..

[3] Derlet R.W., Horowitz B.Z. (1995). Cardiotoxic drugs.. Emerg. Med. Clin. North Am..

[4] Rodríguez-Calvo M.S., Rico R., López-Rivadulla M., Suárez-Peñaranda J.M., Muñoz J.I., Concheiro L. (2002). Report of a suicidal digoxin intoxication: A case report.. Med. Sci. Law.

[5] Eyer F., Steimer W., Müller C., Zilker T. (2010). Free and total digoxin in serum during treatment of acute digoxin poisoning with Fab fragments: Case study.. Am. J. Crit. Care.

